# Metallic and Ceramic Materials Integrity—Surface Engineering for Wear, Corrosion and Erosion Prevention

**DOI:** 10.3390/ma17071541

**Published:** 2024-03-28

**Authors:** Mirosław Szala, Mariusz Walczak

**Affiliations:** Department of Materials Engineering, Faculty of Mechanical Engineering, Lublin University of Technology, Nadbystrzycka 36D, 20-618 Lublin, Poland

## 1. Introduction and Scope

The literature systematically describes the wear behavior and phenomena responsible for the degradation resistance of materials. Overall, wear, corrosion and erosion are the dominant types of engineering material degradation among the broad range of deterioration processes [1,2,3]. Even though the relevant literature [4,5] explains the general factors influencing the resistance of materials, the continuous development of metal-based structure fabrication, processing, treatment technology and surface engineering demands systematic reporting on the advances in the wear properties of metallic and ceramic materials. From both scientific and engineering perspectives, the wear of machine components must be minimized to improve their reliability [6,7,8]. The engineering industry is demanding ceramic- and metal-based structures that perform well in terms of wear, corrosion and erosion environments or optimally in all [9,10,11]. First, to manage that task, material wear mechanisms should be understood [12,13,14]. Computer simulation, numerical calculations or artificial neural networks can be employed to facilitate the selection and design of wear-resistant materials [15,16,17]. Therefore, papers containing experimental and numerical results combined with the effect of material properties on wear, erosion and corrosion resistance are presented in this Special Issue.

The goal of this Special Issue is to complete original studies and review papers related to wear, corrosion and erosion resistance, and the wear mechanisms of metal-based structures and ceramic materials or MMC composites: metal alloys, sinters, hardfacings, thermally sprayed deposits, thin films, composites, additive manufactured metal structures, etc. Papers focused on wear improvement via the modification of microstructural properties, surface layer treatment and the deposition of wear-resistant coatings onto a metal-based substrate were selected.

## 2. Contributions

This Special Issue was open for submissions and welcomed original research contributions and review articles highlighting recent advances and future directions in the fields of wear, corrosion and erosion behaviors of metallic and ceramic structures. It consists of twelve scientific papers, mainly covering the operational performance and properties of metallic and ceramic materials. Thus, in order of publication date the following papers are included.

Świetlicki et al. [18] review the effects of shot peening and cavitation peening on the properties of the surface layer of metallic materials. Pawlik et al. [19] studied the influence of linear energy/heat input coefficient on hardness and weld bead geometry in chromium-rich stringer GMAW coatings. Walczak et al. [20] characterized the corrosion resistance and hardness of shot-peened X5CrNi18-10 steel. Grenadyorov et al. [21] studied the properties of TiAlN coatings obtained by dual-HiPIMS with short pulses. Özkan et al. [22] investigated the effect of AISI H13 steel substrate nitriding on the wear and friction behaviors of AlCrN, ZrN, TiSiN and TiCrN multilayer PVD coatings at different temperatures. Wang et al. [23] researched the effect of grain size on the tribological behavior of the CoCrFeMnNi high-entropy alloy. Xiang et al. [24] studied the impact of marine atmospheric corrosion on the microstructure and tensile properties of a 7075 high-strength aluminum alloy. Yang et al. [25] researched the corrosion and mechanical behavior of the as-cast and solid-solution-treated AM50 magnesium alloy in different media. Chabak et al. [26] investigated the abrasive wear behavior of hybrid high-boron multi-component alloys, including the effect of boron and carbon contents, employing the factorial design method. Purba et al. [27] studied the three-body abrasive wear resistance of 5V/5Nb-5Cr-5Mo-5W-5Co-Fe multicomponent cast alloys with different carbon percentages. Gong et al. [28] investigated the effects of the pre-anodized film thickness on the growth mechanism of plasma electrolytic oxidation coatings on a 1060 Al substrate. Petrunin et al. [29] researched the organosilicon self-assembled surface nanolayers on zinc formation, and their influence on the electrochemical and corrosion of zinc. Finally, Walczak et al. [30] outlined the shot peening effect on sliding wear in 0.9% NaCl of an additively manufactured 17-4PH steel. 

It is worth adding that the Special Issue successfully attracted great interest from authors and readers. Therefore, we will continue to study this field by compiling a second volume entitled “Metallic and Ceramic Material Integrity—Surface Engineering for Wear, Corrosion and Erosion Prevention (2nd Edition)”, which was initiated by the first paper of Okuniewski et al. [31] reviewing the effects of shot peening treatment on the properties of an additively and conventionally manufactured Ti6Al4V alloy. We hope that the second edition also attracts many good-quality research papers.

## 3. Conclusions

The content of this Special Issue is addressed to a broad group of scientists and engineers working in the field of the wear prevention of machine parts and components manufactured with metallic materials. Papers focused on wear improvement via microstructural property modification, surface layer treatment and the deposition of wear-resistant coatings onto a metal-based substrate were included. These papers focus on the experimental aspects of the integrity of metallic and ceramic materials. The performance of metal-based structures, such as conventional metal alloys, hardfacings, coatings, composites and cast metal structures, was studied. The great interest from authors and readers confirms a need for experimental data in the field of surface engineering to understand the physical phenomena involved in the wear and operation performance of engineering materials.

The scientific papers contained in this Special Issue provide new knowledge in the field of surface engineering.
